# Selective Metal Ion Utilization Contributes to the Transformation of the Activity of Yeast Polymerase η from DNA Polymerization toward RNA Polymerization

**DOI:** 10.3390/ijms21218248

**Published:** 2020-11-04

**Authors:** Eva Balint, Ildiko Unk

**Affiliations:** Institute of Genetics, Szeged Biological Research Centre, H-6726 Szeged, Hungary; balint.eva@brc.hu

**Keywords:** polymerase η, enzyme kinetics, yeast, manganese

## Abstract

Polymerase eta (Polη) is a translesion synthesis DNA polymerase directly linked to cancer development. It can bypass several DNA lesions thereby rescuing DNA damage-stalled replication complexes. We previously presented evidence implicating *Saccharomyces cerevisiae* Polη in transcription elongation, and identified its specific RNA extension and translesion RNA synthetic activities. However, RNA synthesis by Polη proved rather inefficient under conditions optimal for DNA synthesis. Searching for factors that could enhance its RNA synthetic activity, we have identified the divalent cation of manganese. Here, we show that manganese triggers drastic changes in the activity of Polη. Kinetics experiments indicate that manganese increases the efficiency of ribonucleoside incorporation into RNA by ~400–2000-fold opposite undamaged DNA, and ~3000 and ~6000-fold opposite TT dimer and 8oxoG, respectively. Importantly, preference for the correct base is maintained with manganese during RNA synthesis. In contrast, activity is strongly impaired, and base discrimination is almost lost during DNA synthesis by Polη with manganese. Moreover, Polη shows strong preference for manganese during RNA synthesis even at a 25-fold excess magnesium concentration. Based on this, we suggest that a new regulatory mechanism, selective metal cofactor utilization, modulates the specificity of Polη helping it to perform distinct activities needed for its separate functions during replication and transcription.

## 1. Introduction

DNA polymerases possess catalytic activity to synthesize DNA in a template-dependent fashion using deoxy-ribonucleoside-triphosphates (dNTPs). However, the attributes of their activities differ considerably reflecting their diverse cellular functions [1,2]. Replicative DNA polymerases are responsible for faithful duplication of the genome and because of that they have a highly selective and restrictive active center ensuring that the correct complementary deoxy-ribonucleoside-monophosphates (dNMPs) are inserted into the growing DNA strand [3,4]. Due to their high selectivity, modifications or lesions in the template strand hinder the movement of replicative DNA polymerases during replication, leading to cell death if unattended. However, translesion synthesis (TLS) DNA polymerases evolved that are capable of synthesizing through DNA lesions [5,6]. These polymerases can take over synthesis from stalled replicative DNA polymerases and carry out synthesis across lesion sites, maintaining the continuity of replication. Contrary to replicative DNA polymerases, the active centers of TLS DNA polymerases are more spacious and less selective, enabling them to accommodate damaged, modified nucleotides. As a result of this, TLS DNA polymerases are error-prone on undamaged DNA, frequently inserting non-complementary nucleosides, which can lead to mutagenesis. Therefore, strict regulatory mechanisms restricting their activities to DNA damage sites are visualized. DNA polymerase η (Polη) is a TLS DNA polymerase that is uniquely able to carry out an efficient and error-free bypass of the most frequent ultraviolet (UV) light-induced DNA lesions, cyclobutane pyrimidine dimers [7]. The importance of this activity is well emphasized by the fact that inactivity of Polη in humans causes the cancer-prone xeroderma pigmentosum variant (XP-V) disorder [8,9]. Polη carries out a mostly error-free bypass of one of the most frequent spontaneous oxidative DNA lesions, 8-oxoguanine (8-oxoG), as well, and it was shown to bypass several other DNA lesions with varying fidelity [5,6,10].

Because of their high fidelity, it was of surprise that replicative DNA polymerases could incorporate ribonucleoside-monophosphates (rNMPs) with relatively high frequency into DNA due to incomplete exclusion of ribonucleoside-triphosphates (rNTPs) from their active centers [11]. Though rNMP incorporation occurs with a much reduced efficiency compared to dNMP, it has been estimated that replicative DNA polymerases incorporate ~10,000 rNMPs into the genome of a yeast cell during a single round of replication, putting rNMPs among the most abundant DNA lesions. The excess presence of rNMPs in DNA went undetected for a long time because they are efficiently removed by ribonucleotide excision repair [12,13]. Beside replicative polymerases, almost all DNA polymerases have been shown to be able to utilize rNMPs during DNA synthesis, though most of them do so with very low efficiencies [14,15,16,17]. However, there are a few exceptions, such as *E. coli* PolV, mycobacterial DinB2, and Polµ, that can utilize rNTPs and dNTPs with comparable efficiencies [18,19,20]. We and others have recently identified the ability of Polη to use rNTPs during synthesis [21,22,23,24]. Akin to other DNA polymerases, Polη incorporates rNMPs during DNA synthesis with a very low efficiency. Even so, experiments with both human and yeast Polη showed that they could contribute to the accumulation of ribonucleosides in the genomes of human and yeast cells [25,26]. Unexpectedly, our results indicated that the RNA synthetic activity of yeast Polη was specific as it inserted rNMPs at least 10-fold more efficiently into RNA over DNA [21]. During RNA extension it could even perform TLS opposite a TT dimer and 8-oxoG in an error-free manner. Moreover, we found that the lack of Polη impaired transcription elongation and caused transcriptional inhibition of several genes. These findings suggested a role for Polη during transcription elongation, and possibly in TLS during transcription. However, we also found that Polη utilized dNTPs with a much higher efficiency than rNTPs during RNA extension. Even though in yeast cells rNTP concentrations are in the millimolar, whereas dNTP concentrations are in the micromolar range, Polη would still synthesize a mixed strand consisting of ribo- and deoxyribonucleosides, which would have detrimental effects on cells. Hence, we surmised that certain cellular factors could improve the specific RNA extension activity of Polη.

DNA polymerases apply a mechanism based on two divalent metal cations during catalysis [27]. The catalytic and nucleotide metal-binding sites at their active center coordinate two metal ions that facilitate the nucleophilic attack by the 3′-OH group of the primer on the α-phosphate of the incoming nucleotide. The presence of a third metal ion at the active center has been recently discovered which is probably needed to reduce the product release barrier [28,29,30]. Mg^2+^ is presumed to be the physiological metal cofactor for DNA polymerases due to its widespread occurrence in nature, its much higher cellular concentration compared with other divalent metal cations, and that in vitro it universally activates DNA polymerases. However, other metal ions such as Mn^2+^, Co^2+^, and Ni^2+^ can be substituted for Mg^2+^ in in-vitro polymerization reactions, but the replacement usually significantly diminishes either the efficiency or the fidelity of the enzyme, or both [31,32]. Notwithstanding, Polβ, Polλ, Polµ, Polι, and PrimPol represent the growing group of exemptions where the activity is improved with the replacement metal. For example, human Polβ exhibited higher reactivity in the presence of Mn^2+^ as compared with Mg^2+^ so that it could even extend a blunt-ended double-stranded DNA template [33]. Kinetic and thermodynamic analysis suggested that Polλ evolved as a Mn^2+^-specific enzyme [34]. Mn^2+^ had positive effects on both the efficiency and the fidelity of gap-filling synthesis by Polµ [35]. Similarly, Polι was more active with Mn^2+^ compared to Mg^2+^ [36]. PrimPol was also found to be a Mn^2+^-dependent enzyme as its DNA primase and polymerase activities, as well its DNA primer/template-binding affinity, significantly improved upon Mn^2+^-binding [37]. Interestingly, the optimal Mn^2+^ concentrations for the above DNA polymerases spanned from the micromolar to the millimolar range. For example, Polι was most active at 75–250 µM Mn^2+^ concentrations on both undamaged and on damage-containing templates [36]. The optimal Mn^2+^ concentrations for Polµ were between 10 and 100 µM during gap-filling and non-homologous end joining [35]. However, Primpol and Polλ were most active around 1, and 1–5 mM Mn^2+^ concentrations, respectively [34,38]

In the present study, we report that substitution of manganese for the metal cofactor magnesium implements drastic changes in the activity of Polη. It greatly impairs its activity and sharply decreases its fidelity during DNA synthesis, whereas RNA synthesis becomes 400–2000-fold more efficient with manganese concomitantly maintaining the base selectivity of the enzyme. Moreover, the weak damage bypass activity of Polη observed during RNA synthesis with magnesium is augmented by 3000–6000-fold with manganese opposite TT dimer and 8-oxoG, respectively. Additionally, of note, manganese is preferred by Polη over magnesium even at a 25-fold lower concentration during RNA synthesis. Based on these findings, we propose a model with a new regulatory mechanism contributing to a shift between the DNA and RNA synthetic activities of Polη.

## 2. Results

### 2.1. Effect of Divalent Metal Ions on the Synthetic Activity of Polη

**Figure 1:**
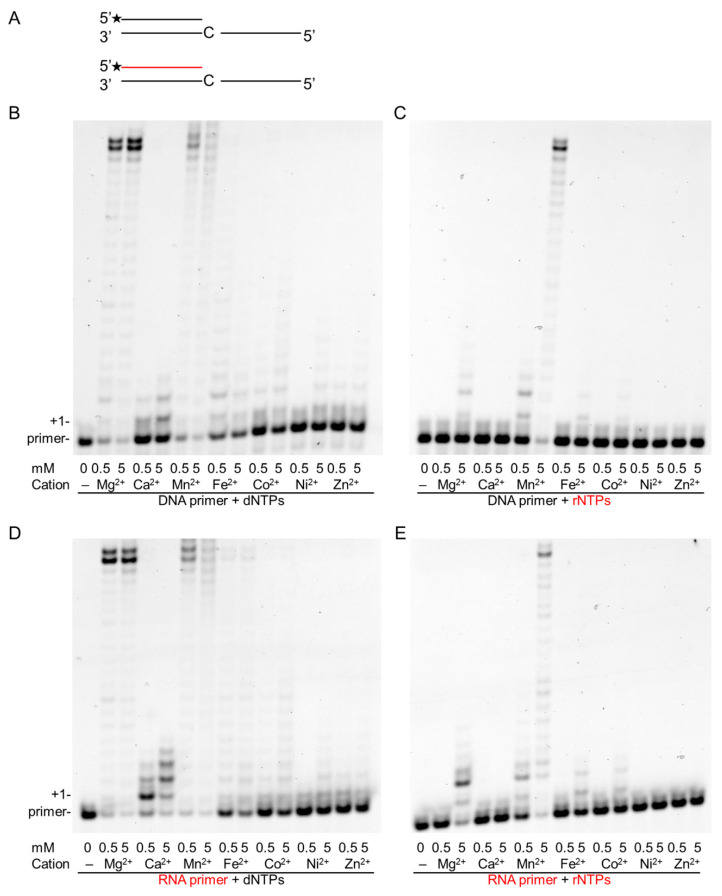
Effect of various divalent cations on the primer extension activities of Polη. In-vitro primer extension reactions were performed with 40-nM Polη for 5 min in the presence of two different concentrations of the indicated divalent cations. (**A**) The structures of the primer/template used in the experiments are shown. The RNA primer is depicted in red. Asterisks (*) indicate fluorescently labeled primer ends. Reactions were carried out in the presence of (**B**) DNA primer and deoxy-ribonucleoside-triphosphates (dNTPs), (**C**) DNA primer with ribonucleoside-triphosphates (rNTPs), (**D**) RNA primer with dNTPs, and (**E**) RNA primer and rNTPs. Reactions in (**B**–**E**) contained 30 nM of the hybridized primer/template, and close to physiological concentrations of nucleotides, either 50 µM of dNTPs or 1 mM of rNTPs. The positions of the primer and its extension by one nucleotide are indicated. RNA primers and rNTPs are highlighted in red.

In our attempt to unravel conditions that could enhance the RNA synthetic activity of Polη, we tested the metal ion dependence of both of its DNA and RNA synthetic activities. Besides magnesium, we compared activities in the presence of six other divalent metal cations that had been implicated in enzymatic activation. In in-vitro primer extension assays, each tested cation supported dNMP insertion into both DNA and RNA primers to a varying extent, although Polη exhibited the highest activity in the presence of Mg^2+^ and Mn^2+^, lengthening almost all the primers and synthesizing until the end of the template (Figure 1B,D). In the case of each metal cofactor, activation could be detected even at low 0.5-mM metal concentrations, whereas a high 5-mM metal concentration resulted in higher activity. Surprisingly, when the reactions were supplemented with rNTPs instead of dNTPs, activity was observed only with Mn^2+^ at a 0.5-mM metal ion concentration using either a DNA or an RNA primer (Figure 1C,E). Moreover, Polη exhibited strong activity only in the presence of Mn^2+^ even at a 5-mM metal concentration, and only a weak activity could be detected with Mg^2+^, Fe^2+^, and Co^2+^, in agreement with our previous results, whereas no activity was detected with the other metals—Ca^2+^, Ni^2+^, and Zn^2+^ [21]. These data suggest that Mn^2+^ could be the proper cation needed for the activation of the RNA synthetic activity of Polη.

### 2.2. Mg^2+^ and Mn^2+^ Concentration-Dependent Synthesis by Polη

**Figure 2:**
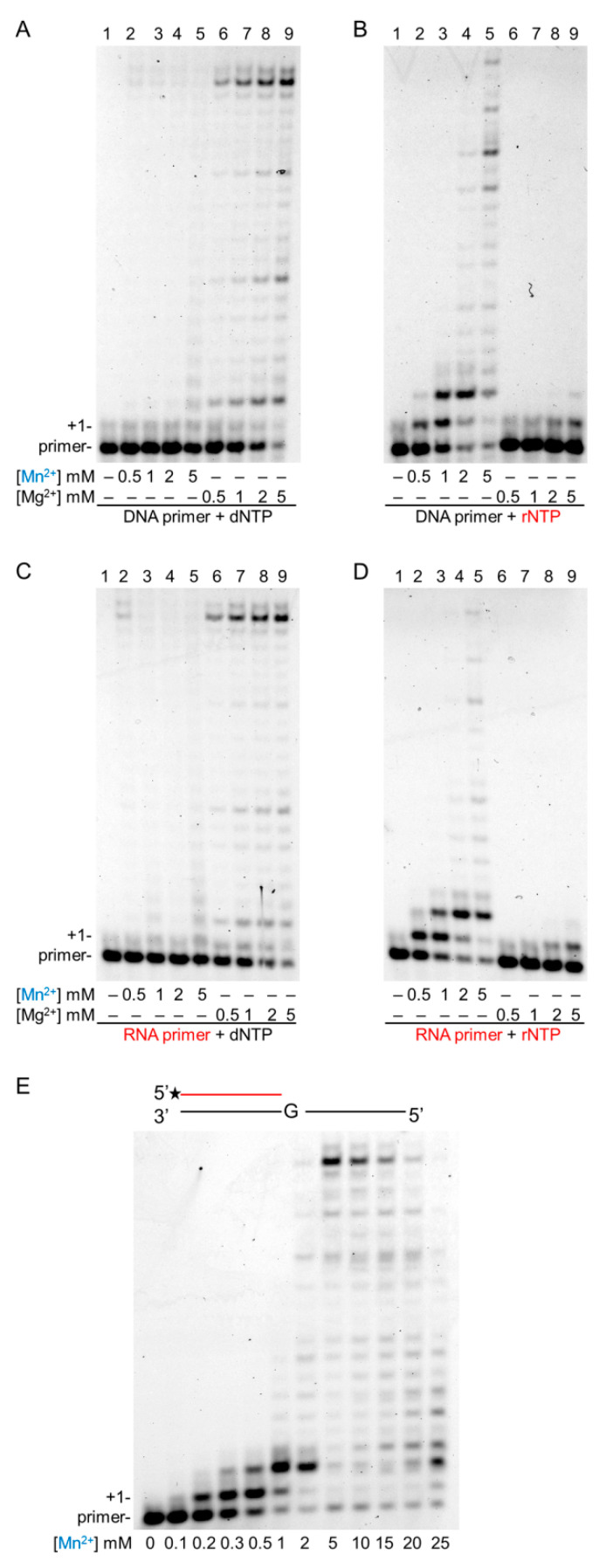
Effect of increasing concentrations of magnesium and manganese ions on the primer extension activity of Polη in the presence of dNTPs or rNTPs. (**A**–**D**) Reactions were performed for 5 min with 10-nM Polη in the presence of 20-nM primer/template, and close to physiological concentrations of nucleotides, either 50-µM dNTPs, or 1-mM rNTPs. (**A**) DNA primer (S3) and dNTPs, (**B**) DNA primer (S3) with rNTPs, (**C**) RNA primer (S7) with dNTPs, and (**D**) RNA primer (S7) and rNTPs. The concentrations of Mn^2+^ and Mg^2+^ are indicated below each panel. Lanes are numbered at the top. (**E**) Optimal manganese concentration needed for the highest activation of Polη during RNA synthesis. Primer extension reactions containing the indicated concentration of Mn^2+^ were performed for 5 min with 20-nM Polη and 1-mM rNTP. The positions of the primer and its extension by one nucleotide are indicated. RNA primers and rNTPs are highlighted in red, and manganese in blue.

To compare the effect of Mg^2+^ and Mn^2+^ on the synthetic activities of Polη, we applied increasing concentrations of the metal ions and carried out synthesis reactions using a DNA or an RNA primer with dNTPs or rNTPs, in all four combinations. In these experiments, the highest synthetic activities were observed at the highest applied (5 mM) metal ion concentrations in all primer–substrate combinations with both Mg^2+^ and Mn^2+^ (Figure 2). As expected, in the presence of a DNA primer and Mg^2+^, Polη exhibited high activity with dNTPs, which sharply increased with increasing Mg^2+^ concentrations (Figure 2A, lanes 6–9). At the highest 5 mM Mg^2+^ concentration, Polη extended ~90% of the primers and synthesis reached the end of the template. Interestingly, a similarly strong activity was observed when the DNA primer was replaced with an RNA primer (Figure 2C, lanes 6–9). On the other hand, substituting Mn^2+^ for Mg^2+^ resulted in greatly diminished dNMP insertions by Polη on both DNA and RNA primers (Figure 2A,C, lanes 1–5). Though a higher Mn^2+^ concentration resulted in a somewhat higher activity, it did not become considerably stronger even at the highest Mn^2+^ concentration, extending only ~30% of the primers. Contrary to the low activation of DNA synthesis, Mn^2+^ dramatically enhanced rNMP insertion by Polη utilizing either a DNA or an RNA primer. Although rNMP incorporation was inefficient with Mg^2+^ extending ~10% of the primers with only 1–2 nucleotides at the highest Mg^2+^ concentration (Figure 2B,D, lanes 6–9), it sharply increased with increasing Mn^2+^ concentration lengthening ~90% of the primers and resulting in fully extended primers at a 5-mM Mn^2+^ concentration (Figure 2B,D, lanes 1–5). The same Mn^2+^ concentration-dependent activation could be detected using individual rNTPs in the assays (Figure S1). In summary, these results showed that although Mn^2+^ strongly reduced the DNA synthetic activity of Polη compared to Mg^2+^, it dramatically elevated its RNA synthetic activity in a concentration-dependent manner.

In order to determine the proper concentration of Mn^2+^ needed for the highest activation of RNA synthesis, we tested reactions containing Mn^2+^ in a broad range of 0.1–25 mM. As Figure 2E shows, Polη was activated at all Mn^2+^ concentrations, and the highest activity was detected with 5-mM Mn^2+^. Lower or higher concentrations resulted in a gradually decreasing activity, though the changes were more moderate at the higher range.

### 2.3. Kinetics of Correct rNMP Incorporation into RNA in the Presence of Mn^2+^

Previously, we determined the kinetic parameters of the RNA synthetic activity of Polη in the presence of Mg^2+^ and found that it incorporated single rNMPs into RNA with an efficiency of ~10^−3^–10^−4^ min^−1^µM^−1^ [21]. To quantitate the enhancement of its RNA synthetic activity observed in the presence of Mn^2+^, we carried out similar steady-state kinetic studies using 5-mM Mn^2+^ instead of 5-mM Mg^2+^ in the reactions (Figure S2). Remarkably, as Table 1 shows, Polη inserted rNMPs into RNA ~1000-fold more efficiently when utilizing Mn^2+^. The smallest ~400-fold increase was detected during rCMP insertion, whereas the highest ~2000-fold difference was measured during rAMP insertion. The overall enhancement was due to a ~100-fold decrease in the Michaelis–Menten constant (K_M_) indicating stronger rNTP-binding of Polη, and to a ~10-fold increase in the catalytic constant (k_cat_) values reflecting the velocity of the reactions. These observed changes in the K_m_ and k_cat_ values indicated a greatly improved specificity of the RNA extension reactions achieved in the presence of Mn^2+^.

### 2.4. The Effect of Mn^2+^ on the Base Selectivity of Polη

**Figure 3:**
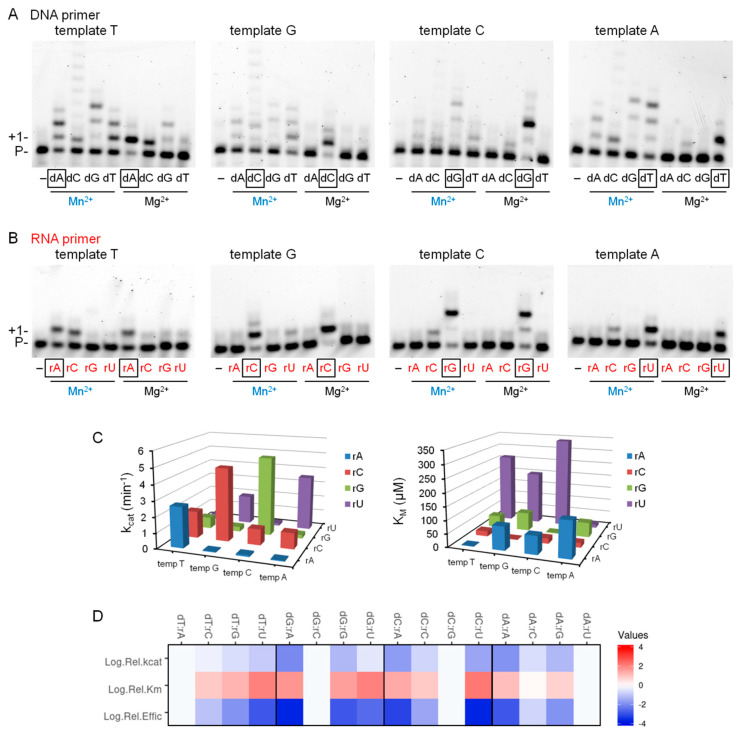
Fidelity of Polη during DNA and RNA synthesis in the presence of magnesium or manganese. (**A**,**B**) Reactions contained 5-mM Mn^2+^ or Mg^2+^ as indicated, 6-nM Polη, 20-nM (**A**) DNA/DNA (S1–4) or (**B**) RNA/DNA (S5–8) primer/template, and (**A**) 0.1-mM individual dNTPs or (**B**) 4 mM individual rNTP, as indicated. Reaction times were 1 min except for the 15-min reactions with Mg^2+^ in (**B**). The templating base in the incoming position is denoted. RNA primer and rNTPs are highlighted in red, and manganese is in blue. The nucleotides representing correct insertions are boxed. (**C**) Measured k_cat_ (left) and K_M_ (right) values of various ribunucleotide incorporations opposite the indicated templating bases. (**D**) Heat map showing relative catalytic constants of incorporation of incorrect versus correct rNTPs. Log.Rel.kcat = Log10(k_cat incorrect_/k_cat correct_), Log.Rel.Km = Log10(K_M incorrect_/K_M correct_) and Log.Rel.Effic = Log10 [(k_cat_/K_M_)_incorrect_/(k_cat_/K_M_)_correct_]. Heat map was generated using http://www.heatmapper.ca [40].

In-vitro Mn^2+^ can be substituted for Mg^2+^ in the activation of several DNA polymerases. However, in most cases the accuracy of DNA synthesis supported by Mn^2+^ drastically decreases, spoiling the activity of the polymerase. Hence, we investigated the effects of Mn^2+^ on the base selectivity of Polη by testing the incorporation of all four dNMPs and rNMPs individually into DNA and RNA primers, respectively, opposite each of the four possible DNA template residues. When DNA synthesis was assayed using Mg^2+^, Polη showed preference for the correct dNTP in accordance with its reported 10^−2^–10^−4^ fidelity (Figure 3A). However, base selectivity was almost completely lost with Mn^2+^ and the correct and incorrect dNMPs were inserted with comparably weak efficiencies. During RNA synthesis with Mg^2+^, Polη discriminated against the incorrect bases catalyzing only weak misinsertions as opposed to robust correct rNMP insertions (Figure 3B). Surprisingly, although Mn^2+^ resulted in a somewhat decreased base selectivity indicated by the stronger intensity of the bands representing misinsertions, a clear preference for the correct rNTPs was maintained. To obtain a more accurate insight on the effect of Mn^2+^ on base selection, we quantitated the fidelity of RNA synthesis in the presence of Mn^2+^ in steady-state kinetic experiments (Figure S2). The results of these studies showed that Polη had a lower affinity to incorrect rNTPs than to correct ones as seen by a ~100-fold increased K_m_ and it incorporated them slower, as seen by a ~10-fold decreased k_cat_ (Table 2 and Figure 3C). Overall, Polη exhibited base selectivity in the 10^−2^–10^−4^ range during RNA synthesis with Mn^2+^ (Table 2 and Figure 3D), which corresponded well with its reported accuracy during DNA synthesis with Mg^2+^ [39]. Interestingly, rAMP misincorporations were the weakest (~10^−3^–10^−4^), whereas rCMP was misinserted with the highest (10^−1^–10^−2^) relative efficiencies opposite all three non-complementary template residues. In summary, the above data indicate that Polη maintained its fidelity during RNA synthesis in the presence of Mn^2+^.

### 2.5. DNA Damage Bypass Activity of Polη with Mn^2+^

**Figure 4:**
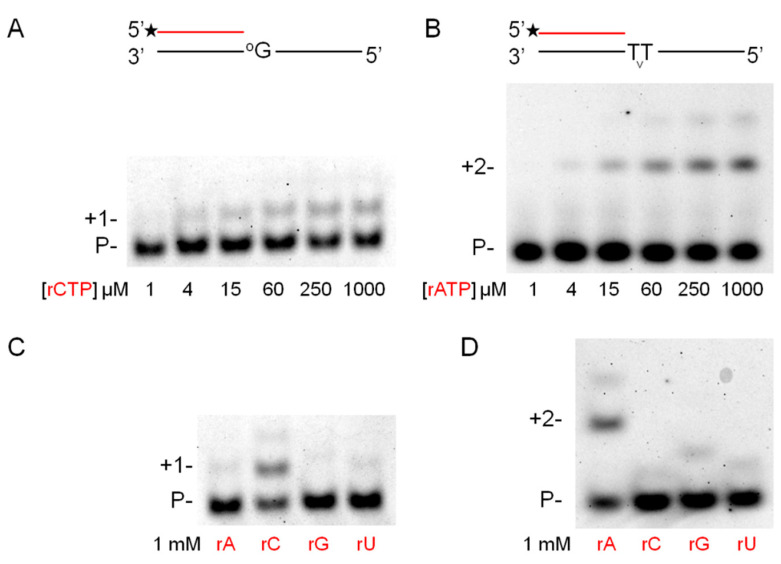
DNA damage bypass by Polη during RNA synthesis in the presence of manganese. (**A**,**B**) The template in S12 contained 8-oxoG in the incoming position. Reactions were performed for 3 min with 5-mM Mn^2+^, 1.6-nM Polη, 8-nM RNA/DNA primer/template and the indicated amount of individual rNTPs. (**C**,**D**) The template in S16 contained TT dimer in the incoming position. Reactions were performed for 15 min with 5-mM Mn^2+^, 1.6-nM Polη, 20-nM RNA/DNA primer/template and the indicated amount of individual rNTPs. RNA primers and rNTPs are highlighted in red.

Next, we examined the effect of Mn^2+^ on the TLS activity of Polη during RNA extension (Figure 4A,B). Our previous results obtained in the presence of Mg^2+^ revealed a very inefficient bypass of 8-oxoG and TT dimer during RNA extension [21]. Importantly, Mn^2+^ had a profound effect on insertion that was opposite to both of these damages (Figure S3). Table 1 shows that a ~6000-fold and a ~3000-fold enhancement in TLS efficiency was measured in steady-state kinetic experiments opposite 8-oxoG and TT dimer, respectively, compared to data obtained in the presence of Mg^2+^. As in the case of undamaged templates and correct incoming nucleotides, the apparent K_m_ values decreased by two orders of magnitude, whereas the K_cat_ values increased by an order of magnitude with Mn^2+^. Moreover, Polη kept its fidelity during the bypass reactions as it preferably inserted the correct rNMPs opposite the damage sites and no significant insertions of the incorrect nucleotides were observed (Figure 4C,D). Based on these results, we concluded that Mn^2+^ was a specific activator of the RNA synthetic activity of Polη both on undamaged templates and opposite DNA damages.

### 2.6. Metal Preference of Polη during RNA Synthesis

**Figure 5:**
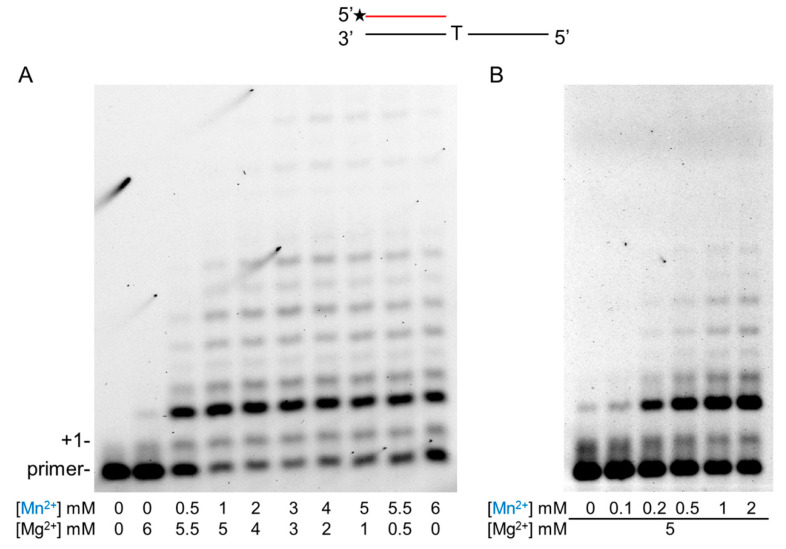
Metal ion preference of Polη during RNA synthesis. Primer extension reactions were performed with 10-nM Polη, 20-nM S6 RNA/DNA primer/template and 1-mM rNTP mix for 5 min. (**A**) Reactions contained both Mn^2+^ and Mg^2+^ in the indicated concentrations. (**B**) Reactions contained 5-mM Mg and the indicated concentrations of Mn^2+^. Manganese is highlighted in blue. The positions of the primer and its one nucleotide extension (+1) are indicated.

Since Mn^2+^ exerted a dramatic effect on Polη activity, it was important to examine which metal cation was preferred by Polη during RNA synthesis. For this reason, we carried out RNA extension experiments with rNTPs in the joint presence of Mg^2+^ and Mn^2+^. In the first set of reactions, the concentration of Mg^2+^ gradually decreased from 6 to 0 mM, whereas the concentration of Mn^2+^ increased from 0 to 6 mM in parallel, maintaining the total metal cation concentration at 6 mM. The pattern of reaction products contrasted strikingly in the sole presence of Mg^2+^ or Mn^2+^ (Figure 5A, compare the first and last lanes), enabling an easy detection of cation utilization. The results showed that, even at eleven-fold excess of Mg^2+^, reaction products specific to Mn^2+^ appeared (Figure 5A second lane). In the next set of reactions, the Mg^2+^ concentration was kept at 5 mM and Mn^2+^ concentration was gradually increased. In this setup, the reaction products showed a Mn^2+^-specific pattern already at a 0.2-mM Mn^2+^ concentration despite the 25-fold higher Mg^2+^ level indicating that Polη preferred Mn^2+^ over Mg^2+^ in the reactions (Figure 5B, lane 3).

## 3. Discussion

The aim of the present study was to identify cellular factors that could improve the RNA synthetic activity of yeast Polη. The conception was based on our previous results showing that Polη has a specific RNA synthetic activity inserting rNMPs at least ten times more efficiently into an RNA primer as opposed to a DNA primer [21]. Despite its specificity, the observed efficiency of RNA synthesis was rather weak raising the possibilities that either the applied reaction conditions were not appropriate or Polη required accessory proteins for efficient RNA synthesis, or both.

Hence, first we tried to optimize the reaction by replacing the generally used metal cofactor Mg^2+^ with other divalent metal cations. We tested several metal cations and all could activate DNA synthesis to varying degrees, but Mg^2+^ and Mn^2+^ achieved the highest activity. On the other hand, during RNA synthesis Ca^2+^, Ni^2+^, and Zn^2+^ were inactive, Mg^2+^, Fe^2+^, and Co^2+^ conferred very limited activity, and only Mn^2+^ supported an efficient reaction. These results suggest that Mn^2+^ is the proper metal cofactor of Polη during RNA synthesis and that other metal cations cannot be substituted for it. Steady-state kinetic experiments revealed that in comparison with Mg^2+^, Mn^2+^ caused a 400–2000-fold increase in efficiency during RNA synthesis on undamaged templates, and a 6000- and 3000-fold increase opposite 8-oxoG and TT dimer, respectively. The specificity of the activation is underpinned by the fact that the enzyme maintained its base selectivity in the 10^−2^–10^−4^ range with Mn^2+^, similarly to its base discrimination during DNA synthesis with Mg^2+^ [39]. Moreover, Polη preferentially utilized Mn^2+^ even in a 25-fold excess of Mg^2+^ during RNA synthesis. Taken together these data reinforce our previous finding that the RNA synthetic activity of Polη is specific, and identify Mn^2+^ as its apposite metal cofactor. Most importantly, our experiments also demonstrate that selective utilization of the two metal cations Mg^2+^ and Mn^2+^ results in a strong difference in specificity. When utilizing Mg^2+^, Polη is proficient in DNA synthesis but very inefficient during RNA synthesis, and preference for the correct base is sustained in both cases. On the other hand, contrary to the remarkable enhancement of RNA synthesis by Mn^2+^, it adversely affected the DNA synthetic activity of Polη by strikingly decreasing both the efficiency and the fidelity of the reaction. This differential effect of Mn^2+^ on the DNA and RNA synthetic activities of Polη sharply contrasts with the effect it had on the reported Mn^2+^-dependent polymerases, Pols λ, ι, µ, and Primpol, in which case Mn^2+^ exerted an overall positive effect on synthesis with either dNTPs or rNTPs [34,35,36,37]. The advantage of enhanced dNMP incorporation is obvious during DNA synthesis. Nevertheless, as it was suggested, increased rNTP utilization could also be advantageous during the repair of DNA double-strand breaks by non-homologous end joining given the easy accessibility of rNTPs. During transcription, however, the DNA synthetic activity of Polη has to be repressed and the RNA synthetic activity has to be elevated to avoid excess dNMP insertion into RNA, which could hinder elongation, lead to miscoding, or otherwise could alter key steps of transcription and translation [41,42,43,44,45,46].

Yet, we have to consider that Mg^2+^ is the most abundant divalent metal cation in the cell. The intracellular concentration of Mg^2+^ is in the millimolar range, which is much higher than the concentration of Mn^2+^ which is in the micromolar range [47]. Therefore, Mg^2+^ could be readily acquired by a plethora of enzymes including Polη during DNA replication. In turn, though our results indicate that Mn^2+^ is preferred over Mg^2+^ by Polη during RNA synthesis even at a ~25-fold lower concentration, given the huge difference between the intracellular concentrations of the two metal ions the involvement of additional factors assisting Mn^2+^-binding has to be presumed. We hypothesize that direct interactions with the transcription machinery could have an effect on the metal utilization of Polη so that Mn^2+^ would be preferred over other cations. One possible way to achieve this could be through direct metal delivery by a metal chaperone, which has been described for many enzymes [48,49]. For example, the metal chaperone copper chaperone for Sod1 (Ccs1) activates the superoxide dismutase Sod1 by directly transferring copper to Sod1, and similarly Cox17 conveys copper to Cox11 for eventual transfer to cytochrome C oxidase for activation [50,51]. Further experiments are needed to unravel the identity of factors that can influence the metal selectivity of yeast Polη and the mechanism of their action.

**Figure 6:**
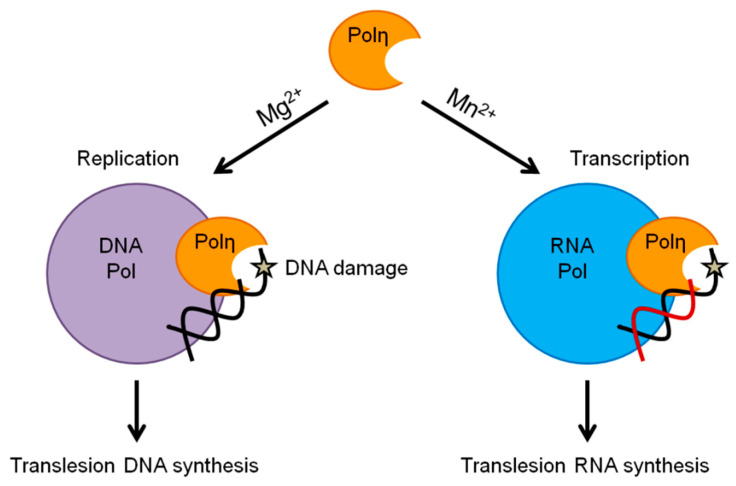
Proposed function of the selective metal cation-dependent activities of yeast Polη. The arrows next to Mg^2+^ and Mn^2+^ symbolize specific strong enhancement of the DNA or RNA synthetic activities of Polη, respectively.

In conclusion, we propose that preferential activation of the DNA or RNA synthetic activity with concomitant impairment of the other through selective metal utilization constitutes a new regulatory mechanism that, with the contribution of other yet unidentified factors, enables Polη to take part in synthesis and DNA damage bypass during replication and also during transcription (Figure 6). Future studies with other polymerases and other enzymes would be necessary to determine the generality of the mechanism. However, since yeast and human Polη exhibit very similar biochemical characteristics during DNA synthesis including processivity, fidelity, damage bypass ability, and human Polη was also shown to be able to utilize rNTPs during DNA extension [23,24,25], therefore it would be of high significance to investigate whether human Polη also has Mn^2+^-activated specific RNA synthetic and translesion RNA synthesis activities, adding an additional layer to its contribution to genome stability.

## 4. Materials and Methods

### 4.1. Protein Purification

*Saccharomyces cerevisiae* Polη was overexpressed in yeast in N-terminal fusion with GST and affinity purified on glutathione–Sepharose beads (GE Healthcare, Uppsala Sweden). as described previously [21]. The GST-tag was removed in the last step of the purification by incubating the beads with PreScission protease (Merck KGaA, Darmstadt, Germany). Efficiency of the purification was verified by polyacrylamide gel electrophoresis and Coomassie staining (Merck KGaA, Darmstadt, Germany).

### 4.2. Oligonucleotides and Primer Extension Assays

Sequences of DNA/DNA and RNA/DNA primer/template substrates used in this study are shown in Table S1. Oligonucleotides used as primers contained a fluorophore indocarbocyanine (Cy3) label at the 5′-ends. Oligonucleotides used in these experiments were purchased from Integrated DNA Technologies, Coralville, Iowa, USA, except for the 8-oxoG-containing primer which was from Midland Certified Reagent Co., Midland, Texas, USA and the TT dimer-containing oligonucleotide was from Trilink Biotechnologies, San Diego, California, USA. Results were also verified with DNA and RNA primers purchased from Sigma-Aldrich Merck KGaA, Darmstadt, Germany. Standard primer extension reactions (5 μL) contained 25-mM Tris/HCl pH 7.5, 1-mM dithiothreitol, 100-μg/mL bovine serum albumin, 10% glycerol, the specified divalent cation as chloride salt, and substrate and enzyme as described in the figure legends. Reactions were initiated by the addition of the cation at the indicated concentrations, incubated at 30 °C and quenched by the addition of 15-μL loading buffer containing 95% formamide, 18-mM EDTA, 0.025% SDS, 0.025% bromophenol blue and 0.025% xylene cyanol. The reaction products were resolved on 10–14% polyacrylamide gels containing 7-M urea and analyzed with a Typhoon TRIO Phosphorimager (GE Healthcare, Little Chalfont, Buckinghamshire, UK).

### 4.3. Determination of Steady-State Kinetic Parameters

Primer extension reactions were performed as described above with the following modifications. On undamaged templates, 1-nM Polη was incubated with 20-nM of primer substrate in standard buffer containing 5-mM Mn. Reactions were initiated by adding the corresponding single rNTP (varied from 0.05 to 500 µM) and incubated at 30 °C from 30 s to 2 min. For kinetic analysis of 8-oxoG or TT dimer bypass, 1-nM Polη was incubated with 8- or 16-nM primer substrate, respectively, in standard buffer containing 5-mM Mn. Reactions were initiated by adding rCTP (0.05 to 500 µM) or rATP (0.05 to 500 µM), and incubated at 30 °C for 3 and 10 min, respectively. The intensity of the gel bands corresponding to the substrate and the product were quantitated with Typhoon TRIO Phosphorimager (GE Healthcare, Little Chalfont, Buckinghamshire, UK) using ImageQuant TL software (version 7.0, GE Healthcare, Little Chalfont, Buckinghamshire, UK) and the observed rates of nucleotide incorporation were plotted as a function of rNTP concentration. The data were fit by non-linear regression using the SigmaPlot program (version 12.5 Systat Software, San Jose, CA, USA) to the Michaelis–Menten equation describing a hyperbola, *v* = *V*max×[rNTP]/(*Km* + [rNTP]. The *k_cat_* and *K_m_* steady-state parameters were obtained from the fit and were used to calculate the efficiency (k_cat_/K_m_) and the relative efficiency (activation by Mn^2+^ versus Mg^2+^) using the formula f_rel_ = (k_cat_/K_m_)_Mn2+_/(k_cat_/K_m_)_Mg2+_. A heat map was generated using http://www.heatmapper.ca [40].

## Figures and Tables

**Table 1:** Comparison of the kinetic parameters of rNTP incorporation into RNA by Polη using Mg^2+^ or Mn^2+^ as cofactors.

Templating Nucleotide	Incoming Nucleotide	Cation	k_cat_ (min^−1^)	K_M_ (µM)	k_cat_/K_M_ (min^−1^µM^−1^)	Relative Efficiency ^a^
T	rATP	Mg^2+^	0.24 ± 0.01 ^b^	466 ± 47.3 ^b^	5.15 × 10^−4 b^	
		Mn^2+^	2.61 ± 0.14	2.51 ± 0.64	1.04	2019
G	rCTP	Mg^2+^	2.76 ± 0.06 ^b^	438 ± 37.5 ^b^	6.30 × 10^−3 b^	
		Mn^2+^	4.68 ± 0.22	1.89 ± 0.42	2.48	394
C	rGTP	Mg^2+^	0.45 ± 0.01 ^b^	394 ± 52 ^b^	1.14 × 10^−3 b^	
		Mn^2+^	5.07 ± 0.27	2.55 ± 0.63	1.99	1746
A	rUTP	Mg^2+^	0.10 ± 0.01 ^b^	423 ± 90.4 ^b^	2.36 × 10^−4 b^	
		Mn^2+^	3.51 ± 0.19	12.8 ± 2.25	2.74 × 10^−1^	1161
8-oxo-G	rCTP	Mg^2+^	0.034 ± 0.004^b^	974 ± 270 ^b^	3.52 × 10^−5^	
		Mn^2+^	0.275 ± 0.01	1.25 ± 0.28	2.20 × 10^−1^	6286
TT dimer	rATP	Mg^2+^	0.0083 ± 0.001 ^b^	1678 ± 445 ^b^	4.94 × 10^−6^	
		Mn^2+^	0.174 ± 0.005	11.3 ± 1.35	1.54 × 10^−2^	3117

^a^ Relative efficiency was calculated using the following equation: f_rel_ = (k_cat_/K_M_)_Mn2+_/(k_cat_/K_M_)_Mg2+_. ^b^ Published in [21].

**Table 2:** Kinetic parameters of rNTP incorporation and misincorporation into RNA by Polη using Mn^2+^ as cofactor.

Templating Nucleotide	Incoming Nucleotide	k_cat_ (min^−1^)	K_M_ (µM)	k_cat_/K_M_ (min^−1^µM^−1^)	Relative Efficiency ^a^	Discrimination1/f ^b^
T	rATP	2.61 ± 0.14	2.51 ± 0.64	1.04		
	rCTP	1.72 ± 0.06	19.4 ± 3.14	0.09	8.7 × 10^−2^	12
	rGTP	0.72 ± 0.03	44.6 ± 7.03	0.016	1.5 × 10^−2^	67
	rUTP	0.36 ± 0.02	261 ± 50.6	0.0014	1.3 × 10^−3^	769
G	rATP	0.06 ± 0.00	89.3 ± 17.7	0.0007	2.8 × 10^−4^	3571
	rCTP	4.68 ± 0.22	1.89 ± 0.42	2.48		
	rGTP	0.31 ± 0.02	68.9 ± 14.9	0.0045	1.8 × 10^−3^	555
	rUTP	1.86 ± 0.04	199 ± 14.5	0.0093	3.8 × 10^−3^	263
C	rATP	0.10 ± 0.00	69.2 ± 11.9	0.0014	7.0 × 10^−4^	1429
	rCTP	1.03 ± 0.04	19.9 ± 3.74	0.052	2.6 × 10^−2^	38
	rGTP	5.07 ± 0.27	2.55 ± 0.63	1.99		
	rUTP	0.16 ± 0.01	339 ± 49.4	0.0005	2.5 × 10^−4^	4000
A	rATP	0.05 ± 0.00	138 ± 26.2	0.0004	1.48 × 10^−3^	676
	rCTP	1.03 ± 0.07	17.5 ± 4.92	0.059	2.2 × 10^−1^	5
	rGTP	0.24 ± 0.01	55.9 ± 8.64	0.0043	1.6 ×10^−2^	63
	rUTP	3.51 ± 0.19	12.8 ± 2.25	0.27		

^a^ Relative efficiency was calculated using the following equation: f_rel_ = (k_cat_/K_M_)_incorrect_/(k_cat_/K_M_)_correct_. ^b^ Inverse relative efficiency: 1/f_rel_ = (k_cat_/K_M_)_correct_/(k_cat_/K_M_)_incorrect._

## Supplementary Materials

The following are available online at https://www.mdpi.com/1422-0067/21/21/8248/s1, Figure S1, Figure S2, Figure S3, Table S1.
