# Supplementary material for: Selective Metal Ion Utilization Contributes to the Transformation of the Activity of Yeast Polymerase η from DNA Polymerization toward RNA Polymerization
Source: Int J Mol Sci. 2020 Nov 4;21(21):8248. doi: 10.3390/ijms21218248 (PMC7672554; doi:10.3390/ijms21218248)
Supplement: Supplementary file 1 [file ijms-21-08248-s001.zip › ijms-983238-supplementary.docx]

**Selective metal ion utilization contributes to the transformation of the activity of yeast polymerase η from DNA polymerization toward RNA polymerization**

**Supplementary**


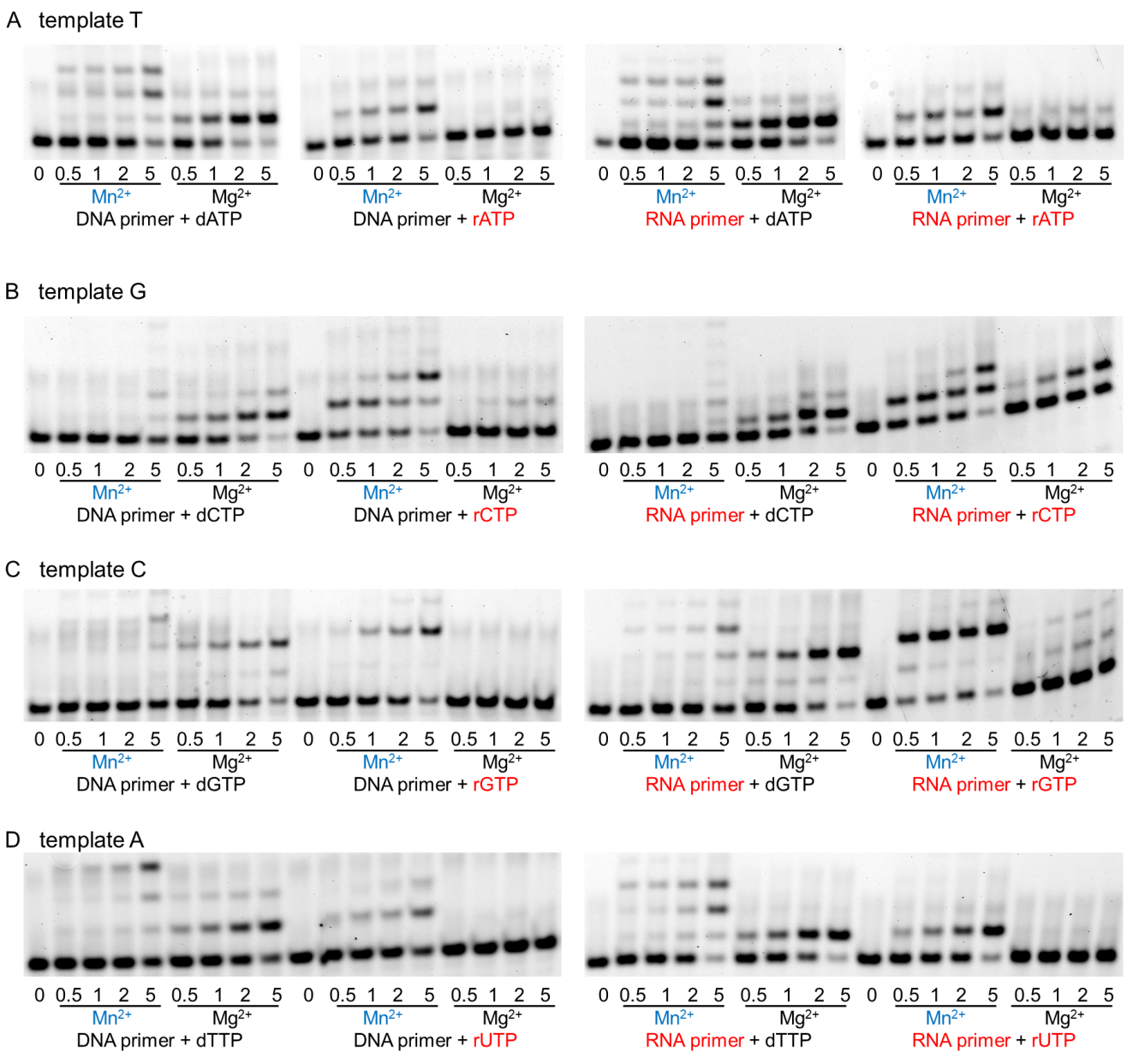


Figure S1. Single ribonucleotide incorporation in the presence of manganese or magnesium. Primer extension reactions containing the indicated concentration of Mn^2+^ or Mg^2+^ were performed for 5 minutes with 6 nM Pol η, 20 nM DNA primer/DNA template (S1-4), or RNA primer/DNA template (S5-8), and 50 μM of the incoming individual correct dNTP or 1 mM of the individual correct rNTP, as indicated below the panels. The templating bases are indicated at the top.


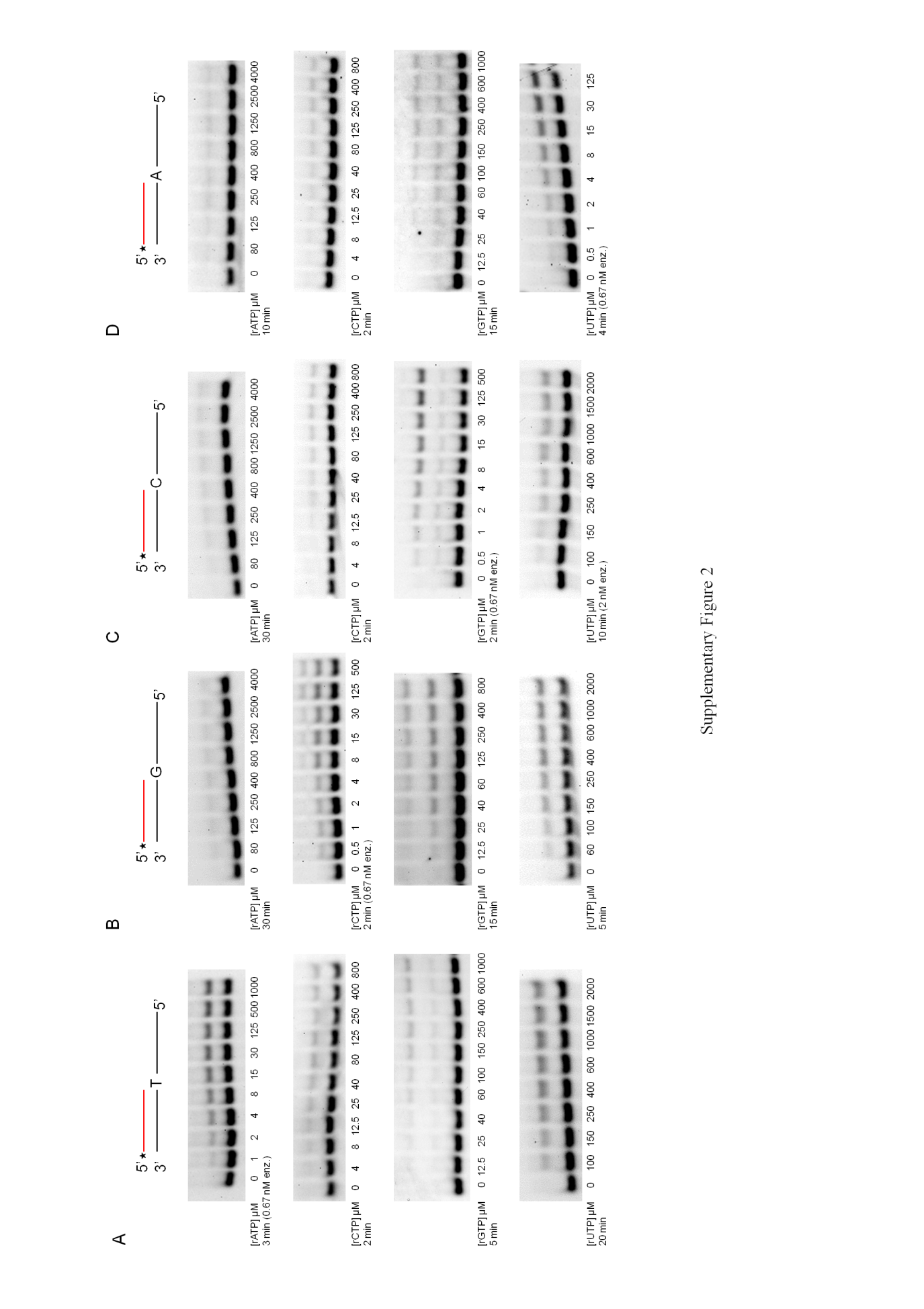


**Figure S2.** Determination of the kinetic parameters of rNTP incorporation and misincorporation into RNA primer by Polη using Mn^2+^ as cofactor. Templates contained T (A), G (B), C (C) or A (D) in the incoming position (S5-8), as denoted above the panels. Reactions contained 5 mM Mn^2+^, 20 nM RNA primer/DNA templare, 1 nM Polη (unless otherwise indicated) and increasing concentration of an individual rNTP as shown. Reaction times are also indicated.


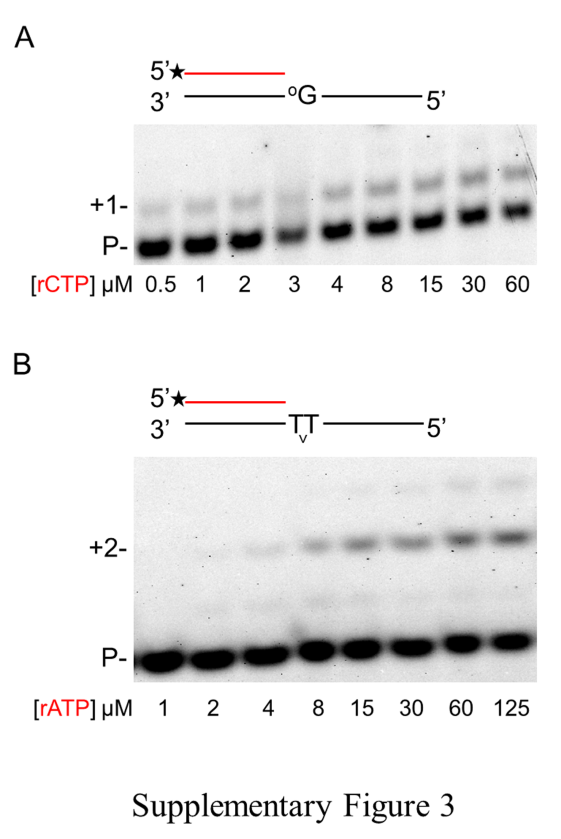


Figure S3. Determination of the kinetic parameters of DNA damage bypass by Polη using RNA primer and Mn^2+^ as cofactor. A) The template (S12) contained 8-oxoG in the incoming position. Reactions in the presence of 5 mM Mn^2+^, 8 nM RNA primer/DNA templare, 0.8 nM Polη and increasing concentration of rCTP, as shown, were performed for 6 min. B) Reactions with S16 containing a TT dimer in the incoming position were performed for 20 min in the presence of 5 mM Mn^2+^, 16 nM RNA primer/DNA templare, 1.6 nM Polη and increasing concentration of rATP, as shown.

Table S1. Sequence and structure of substrates used in the *in vitro* primer extension assays. RNA primers are in red. The Cy3 label at the 5’ end of primers is indicated. The first templating nucleotides are in bold.

| **Substrate** | **Sequence** |
| --- | --- |
| S1 | /5Cy3/CGCTACCTAGCCTGCCTCAAGAGTTGCTCG  3’-GCGATGGATCGGACGGAGTTCTCAACGAGC**A**CAGGCTTACGCTCAGGTCG-5’ |
| S2 | /5Cy3/CGCTACCTAGCCTGCCTCAAGAGTTGCTCG  3’-GCGATGGATCGGACGGAGTTCTCAACGAGC**T**CAGGCTTACGCTCAGGTCG-5’ |
| S3 | /5Cy3/CGCTACCTAGCCTGCCTCAAGAGTTGCTCG  3’-GCGATGGATCGGACGGAGTTCTCAACGAGC**G**CAGGCTTACGCTCAGGTCG-5’ |
| S4 | /5Cy3/CGCTACCTAGCCTGCCTCAAGAGTTGCTCG  3’-GCGATGGATCGGACGGAGTTCTCAACGAGC**C**CAGGCTTACGCTCAGGTCG-5’ |
| S5 | /5Cy3/CGCUACCUAGCCUGCCUCAAGAGUUGCUCG  3’-GCGATGGATCGGACGGAGTTCTCAACGAGC**A**CAGGCTTACGCTCAGGTCG-5’ |
| S6 | /5Cy3/CGCUACCUAGCCUGCCUCAAGAGUUGCUCG  3’-GCGATGGATCGGACGGAGTTCTCAACGAGC**T**CAGGCTTACGCTCAGGTCG-5’ |
| S7 | /5Cy3/CGCUACCUAGCCUGCCUCAAGAGUUGCUCG  3’-GCGATGGATCGGACGGAGTTCTCAACGAGC**G**CAGGCTTACGCTCAGGTCG-5’ |
| S8 | /5Cy3/CGCUACCUAGCCUGCCUCAAGAGUUGCUCG  3’-GCGATGGATCGGACGGAGTTCTCAACGAGC**C**CAGGCTTACGCTCAGGTCG-5’ |
| S12 | /5Cy3/CGACGAUGCUCCGGUACUCCAGUGUAGGCAU  3’-CAAAAGGGTCAGTGCTGCTACGAGGCCATGAGGTCACATCCGTA**^O^G**AATGCTTAA  GAACTCCGTCCGTACCATCGA-5’ |
| S16 | /5Cy3/CGUAUUCGCGCGC  3’-CGAATGGCGGTGCG**T^T**GCGCGCGAATACG-5’ |
